# Deep neural network models for cell type prediction based on single-cell Hi-C data

**DOI:** 10.1186/s12864-024-10764-7

**Published:** 2024-09-16

**Authors:** Bing Zhou, Quanzhong Liu, Meili Wang, Hao Wu

**Affiliations:** 1 School of Software, Shandong University, Jinan, Shandong 250100 China; 2 College of Information Engineering, Northwest A&F University, 712100 Yangling, Shaanxi China

**Keywords:** Deep neural networks, Single-cell Hi-C data, Cell type prediction, Cell classification

## Abstract

**Background:**

Cell type prediction is crucial to cell type identification of genomics, cancer diagnosis and drug development, and it can solve the time-consuming and difficult problem of cell classification in biological experiments. Therefore, a computational method is urgently needed to classify and predict cell types using single-cell Hi-C data. In previous studies, there is a lack of convenient and accurate method to predict cell types based on single-cell Hi-C data. Deep neural networks can form complex representations of single-cell Hi-C data and make it possible to handle the multidimensional and sparse biological datasets.

**Results:**

We compare the performance of SCANN with existing methods and analyze the model by using five different evaluation metrics. When using only ML1 and ML3 datasets, the ARI and NMI values of SCANN increase by 14% and 11% over those of scHiCluster respectively. However, when using all six libraries of data, the ARI and NMI values of SCANN increase by 63% and 88% over those of scHiCluster respectively. These findings show that SCANN is highly accurate in predicting the type of independent cell samples using single-cell Hi-C data.

**Conclusions:**

SCANN enhances the training speed and requires fewer resources for predicting cell types. In addition, when the number of cells in different cell types was extremely unbalanced, SCANN has higher stability and flexibility in solving cell classification and cell type prediction using the single-cell Hi-C data. This predication method can assist biologists to study the differences in the chromosome structure of cells between different cell types.

## Background

The single-cell Hi-C technology verifies that the organization module of single cell is the basis of dynamic chromosomes [1], and reveals that the chromosome conformation diagram can be reinterpreted by cell cycle [2]. Single-cell Hi-C technology is constantly being updated and expanded, such as single-nucleus Hi-C that provides 10 times more contacts per cell than previous methods [3], single-cell combinatorial indexed Hi-C that isolated cells by differences in karyotype and cell cycle status [4], and the Dip-C technology [5]. Therefore, there is a lack of convenient and accurate methods to classify cell types according to the variation of chromosome structure for cells of different cell types.

In previous studies, the contact information difference between two cells was mainly used as similarity to measure the cells, so as to complete the task of Hi-C data analysis [6–9]. And some studies have comprehensively tested the above approach using similarity [10]. For example, HiCRep+MDS has been used to capture meaningful information of the cell cycle from single-cell Hi-C data in a low-dimensional space, but it performed poorly in cell type recognition [11, 12]. Nies et al. [13] comprehensively analyzed the clustering verification methods and four clustering methods, and summarized the advantages and disadvantages of various clustering algorithms. But, there still lacks a stable and flexible method to carry out accurate cell classification and cell type prediction.

Deep learning technology has been extensively studied in different studies, such as image classification [14], speech recognition [15], handwritten text transcription [16], automatic driving [17] and recommendation system [18]. In addition, deep learning technique has been applied to Hi-C data to enhance the resolution of the data [19–21]. In this study, we present a cell classification and cell type prediction method by constructing a deep neural network model named SCANN to classify single-cell Hi-C data. The single-cell Hi-C data is preprocessed by the convolution and restart-random-walk and principal component analysis [4, 12], and the embedding of each cell is generated as the input of the model after a filter step [22]. SCANN applies machine learning and deep learning knowledge to achieve excellent cell classification and cell type prediction for single-cell Hi-C data.

## Results

### Overview of SCANN’s performance

We validate the cell classification performance of SCANN, and evaluate it using low-resolution single-cell Hi-C dataset. Ramani et al. [4] proposed the sciHi-C technology to generate the single-cell Hi-C datasets of six libraries, which comprised a total of 10,696 cells from human cell lines (HeLa, HAP1, GM12878, and K562). To train and test SCANN, we select 2,661 high-quality human cells of all six libraries by quality control, which are detected at least 5k contacts in a chromosome contact matrix.

**Fig. 1:**
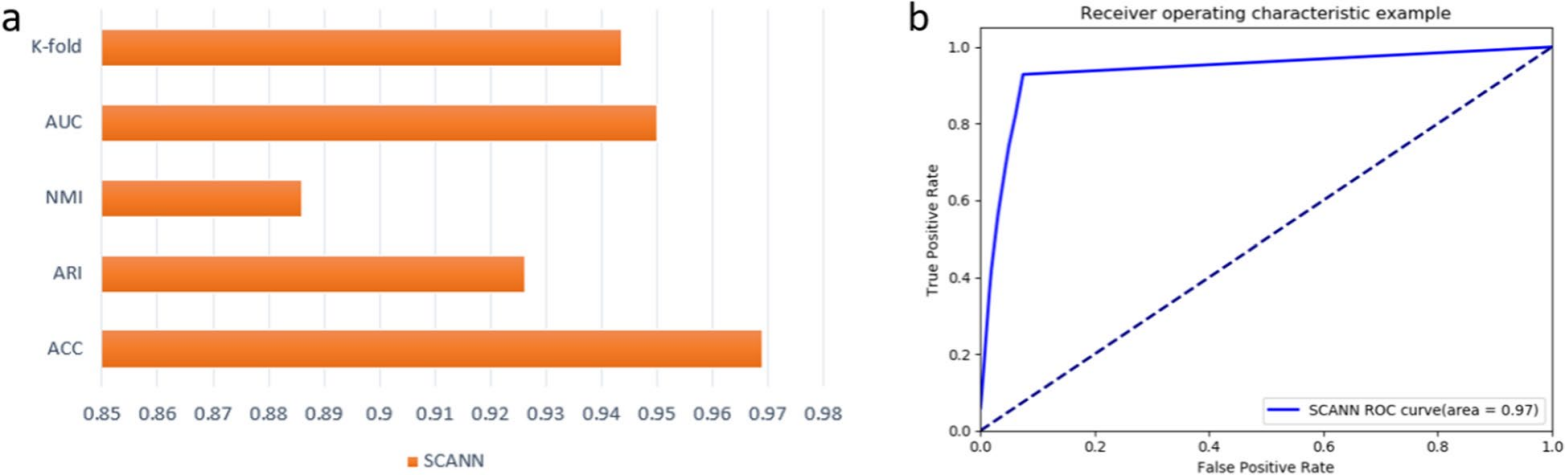
Loss and accuracy curve of SCANN. **a** loss function curve of SCANN as the number of iterations increases, where the dotted line represents the training loss, and the solid line represents the verification loss. **b** accuracy curve of the SCANN as the number of iterations increases, where the dotted line represents the training accuracy, and the solid line represents the verification accuracy

As shown in Fig. 1, SCANN gradually levels off as the loss of training and verification decreases and as the accuracy of training and verification increases. The loss of training is basically stable after the iteration 60, but the loss of verification begins to increase after reaching the minimum value in 10 iterations. As previously suggested, insufficient training dataset might cause the overfitting of the model, so we minimize the increase in the loss value through dropout operation to reduce overfitting degree of the model. Based on this, we determine the number of training samples through the loss curve to get a perfect model finally.

**Fig. 2:**
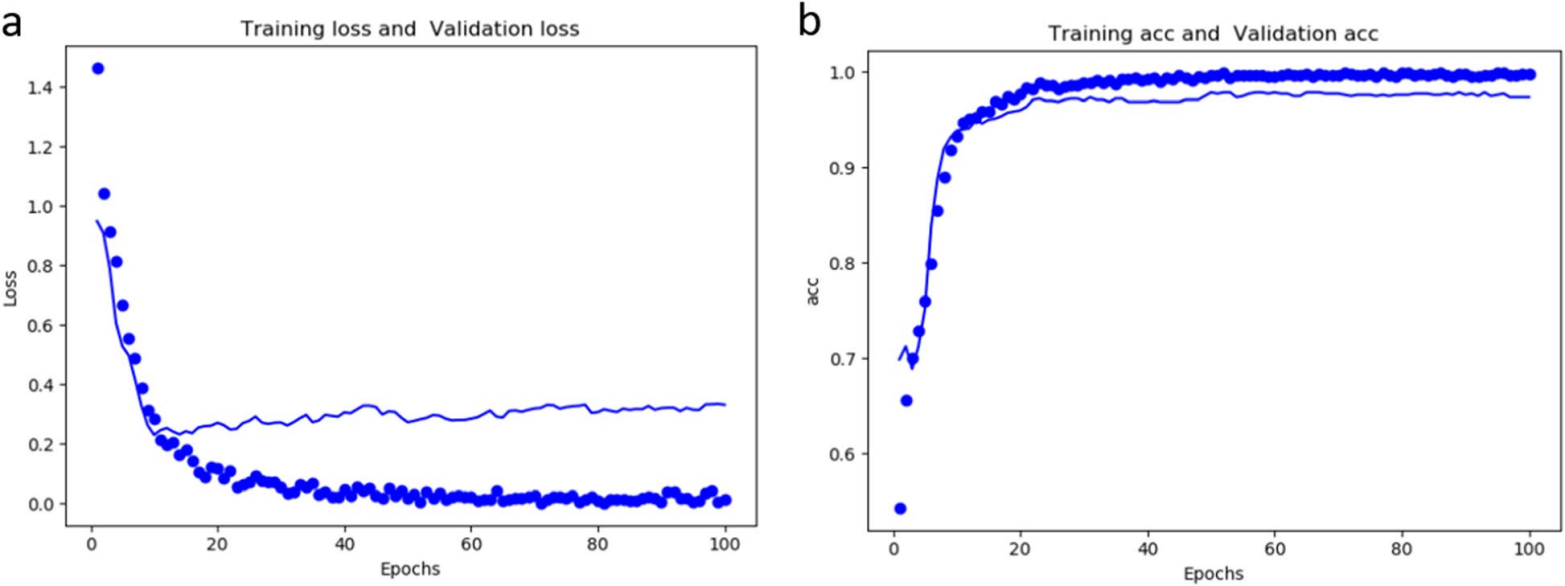
Performance evaluation of SCANN. **a** the values of K-fold, AUC, NMI, ARI, ACC indicators of SCANN, where the abscissa is the index score, and the ordinate is five different measurements. **b** the ROC curves of SCANN, where the abscissa is FP rate, and the ordinate is TP rate

As shown in Fig. 2a, we employ SCANN to predict cell types and evaluate it using five different measures including k-fold, area under curve (AUC), accuracy (ACC), high adjusted rand index (ARI) and normalized mutual information (NMI). The analysis shows that the values of k-fold, AUC, NMI, ARI and ACC are 0.943, 0.95, 0.886, 0.926 and 0.969 respectively.

The result of k-fold indicates that the model can make effective use of an limited data to accomplish the prediction task efficiently. The AUC score indicates that the model can correctly predict the sample type in the case of an unbalanced sample size (Fig. 2b). The values of ARI and NMI show that the prediction results of the model are close to the real results, the cells of the same type in the classification results have high similarity, and the cells among different types have high dispersion. In addition, the ACC score indicates that the model could predict cell types correctly. Combined with the scores of five indicators, SCANN could predict the accurate cell types very from single-cell Hi-C data.

**Fig. 3:**
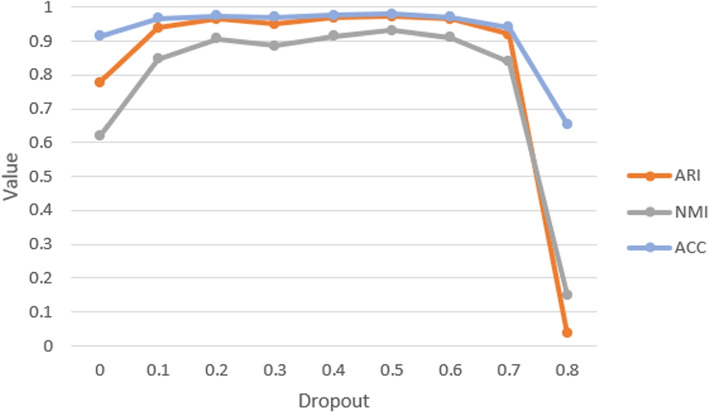
The effect of dropping rate in Dropout on the three prediction indicators

SCANN realizes the function of cell type recognition well and gets high scores on the five different metrics. The training process of SCANN shows the efficient advantage of the deep learning method. And we solve the overfitting problem due to the insufficient training set in this study (Fig. 3). The employment of Dropout method helps to improve the cell prediction ability of the model. Specifically, when the discard rate increases from 0.0 to 0.5, the values of ARI, NMI and ACC increase steadily and peak at 0.5. When the discard rate is 0.8, the values of these three indicators plummet. This is because too many neurons are discarded, resulting in incomplete neural networks that were not sufficient to predict cell type. Moreover, when we take out 50 percent of the neurons in the neural network, the value of ARI increases by 25.1 %, the value of NMI increases by 50.7 %, and the value of ACC increases by 7.1 % compared with that without using Dropout.

### The results on the public single-cell Hi-C dataset

**Fig. 4:**
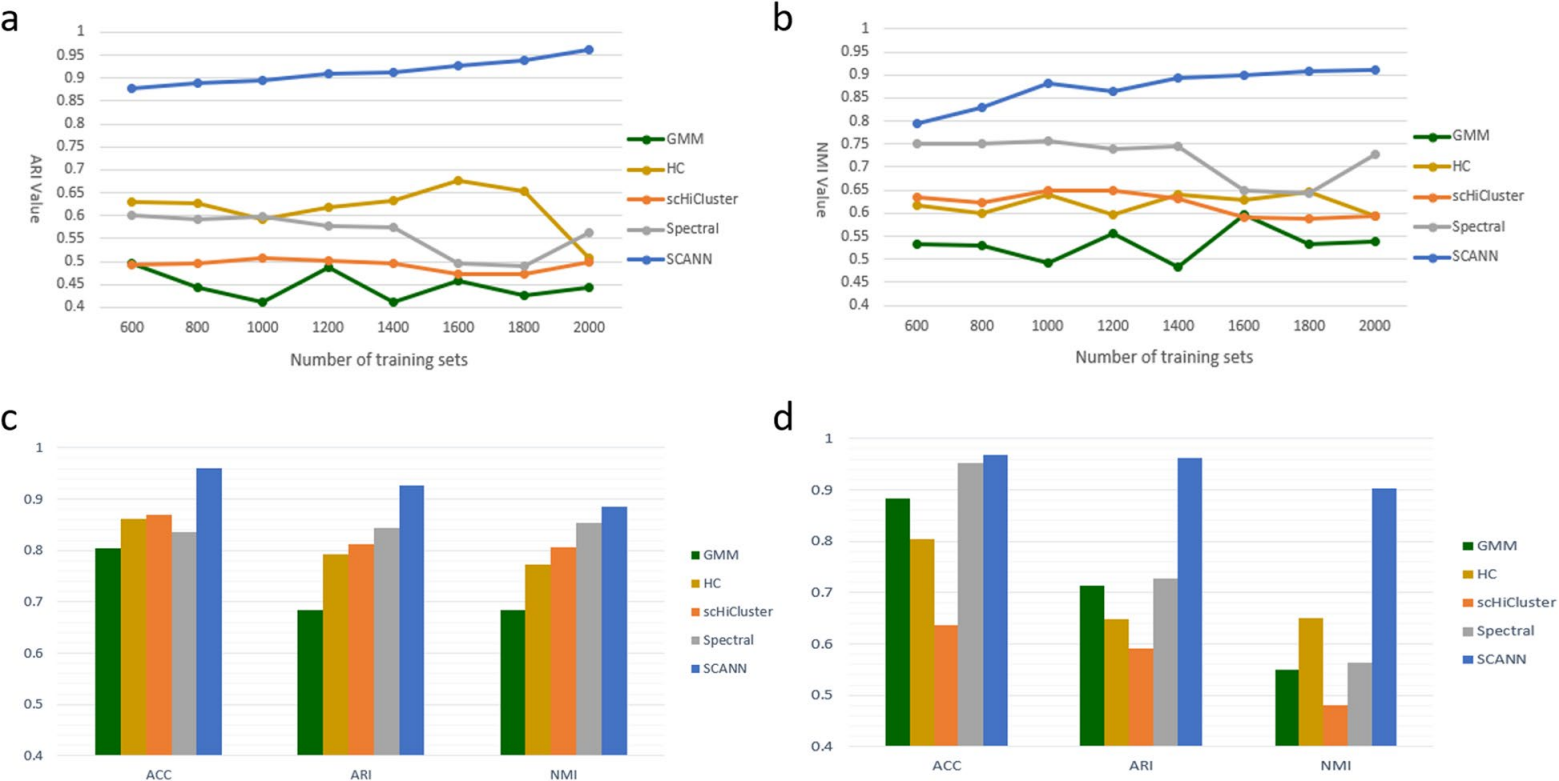
A comparison of different analysis tasks performed by SCANN against four other methods. **a** the ARI values of the model comparing to four other methods, as the amount of data increases. **b** the NMI values of the model comparing to four other methods, as the amount of data increases. **c** when using only ML1 and ML3 libraries, our model compares its performance with the four other methods by their ACC, ARI and NMI scores. **d** when using all six libraries, our model compares its performance with the four other methods by their ACC, ARI and NMI scores

We compare SCANN with four methods: scHiCluster, spectral clustering, Gaussian Mixture Model (GMM) and Hierarchical clustering (HC) in terms of the prediction capability, where scHiCluster approach had achieved clustering of single-cell Hi-C dataset by means of k-means++ algorithm [12], and spectral clustering is a popular clustering algorithm in recent years with simple implementation and better clustering results than traditional methods [23], and researchers had modified and customized hierarchical clustering method to address unique challenges in the analysis of scRNA-SEQ data, such as deletions of low-expression genes [24]. In addition, Yang et al. [25] used GMM to distinguish the molecular subtypes of tumor specimens with the help of generative adversarial networks. As shown in Fig. 4, SCANN performs better than the four methods on the same dataset in terms of model performance and stability against abnormal data.

We compare SCANN with the above four methods on two datasets of different sizes. As shown in Table 1, the first dataset has been used in [12]. The two datasets are both used in our study, which are controlled by the same cell quality control. The ML1 and ML3 libraries have 626 cells, including 44 GM12878 cells, 214 HAP1 cells, 258 HeLa cells and 110 K562 cells. The second dataset contains 2661 cells from all six libraries, of which the cell number of HAP1 and HeLa is 746 and 1759 respectively, and the cell number of GM12878 and K562 is 45 and 111 respectively. This shows that the cell number of HAP1 and HeLa is much higher than that of the other two types of cells, resulting in an uneven distribution of cell number in second dataset.

**Table 1:** The number of cells from all six libraries and only ML1, ML3 libraries

Datasets	GM12878	HAP1	HeLa	K562	Total
ML1, ML3 libraries	44	214	258	110	626
Six libraries	45	746	1759	111	2661

As shown in Fig. 4c and Table 2, when using 626 cells from ML1 and ML3 libraries, scHiCluster and three baseline methods can work well, because the number of cells in different cell types is evenly distributed. Specifically, ACC scores of four other methods are 0.80, 0.86, 0.87 and 0.84 respectively, ARI scores of them are 0.68, 0.79, 0.81 and 0.84 respectively, and NMI scores of them are 0.68, 0.77, 0.80 and 0.85 respectively. However, SCANN obtains better prediction results than four other methods. ACC, ARI and NMI scores of our method are 0.96, 0.92 and 0.89 respectively. This shows that SCANN has optimal performance comparing to the existing methods, as shown by ACC, ARI and NMI indicators.

**Table 2:** The scores of five methods when all six libraries and only ML1 and ML3 libraries are used

	Methods	ARI	NMI	ACC
ML1, ML3 libraries	GMM	0.68	0.68	0.80
	HC	0.79	0.77	0.86
	scHiCluster	0.81	0.80	0.87
	Spectral cluster	0.84	0.85	0.84
	SCANN	0.92	0.89	0.96
Six libraries	GMM	0.71	0.55	0.88
	HC	0.64	0.65	0.80
	scHiCluster	0.59	0.48	0.63
	Spectral cluster	0.72	0.56	0.95
	SCANN	0.96	0.90	0.97

As shown in Fig. 4d and Table 2, when used on the 2661 cells from all six libraries, the scHiCluster and HC obtain poor cell classification results. One of the possible reasons is a great difference in the number of cells among different cell types in the second dataset. However, our method achieves better performance using all six libraries than only ML1 and ML3 libraries (the number of samples increases more than fourfold). Specifically, ACC scores of four other methods are 0.88, 0.80, 0.63 and 0.95 respectively. ARI scores of them are 0.71, 0.64, 0.59 and 0.72 respectively, and NMI scores of them are 0.55, 0.65, 0.48 and 0.56 respectively. However, ACC, ARI and NMI scores of SCANN are 0.97, 0.96 and 0.90 respectively. This is not only a significant improvement comparing to the scHiCluster and three baseline methods, but also better than the results obtained when using ML1 and ML3 libraries.

Experimental results show that SCANN is superior to four other methods in different aspects. This is especially the case when used to larger dataset. In addition, our method is robust even if there is a big difference in the number of cells among different cell types.

As shown in Fig. 4a & b, the ability of SCANN on predicting cell type improves with the increase of data quantity. The larger the dataset, the better the results of SCANN. When the number of training samples is 2000 and the number of test samples is 661, as shown in Fig. 4a, ARI score of SCANN increases by 89.3% over that of hierarchical clustering method and increases by 71.0% over that of spectral clustering method. In addition, NMI score of SCANN increases by 53.2% over that of scHiCluster algorithm and increases by 25.3% over that of spectral clustering method (Fig. 4b).

**Fig. 5:**
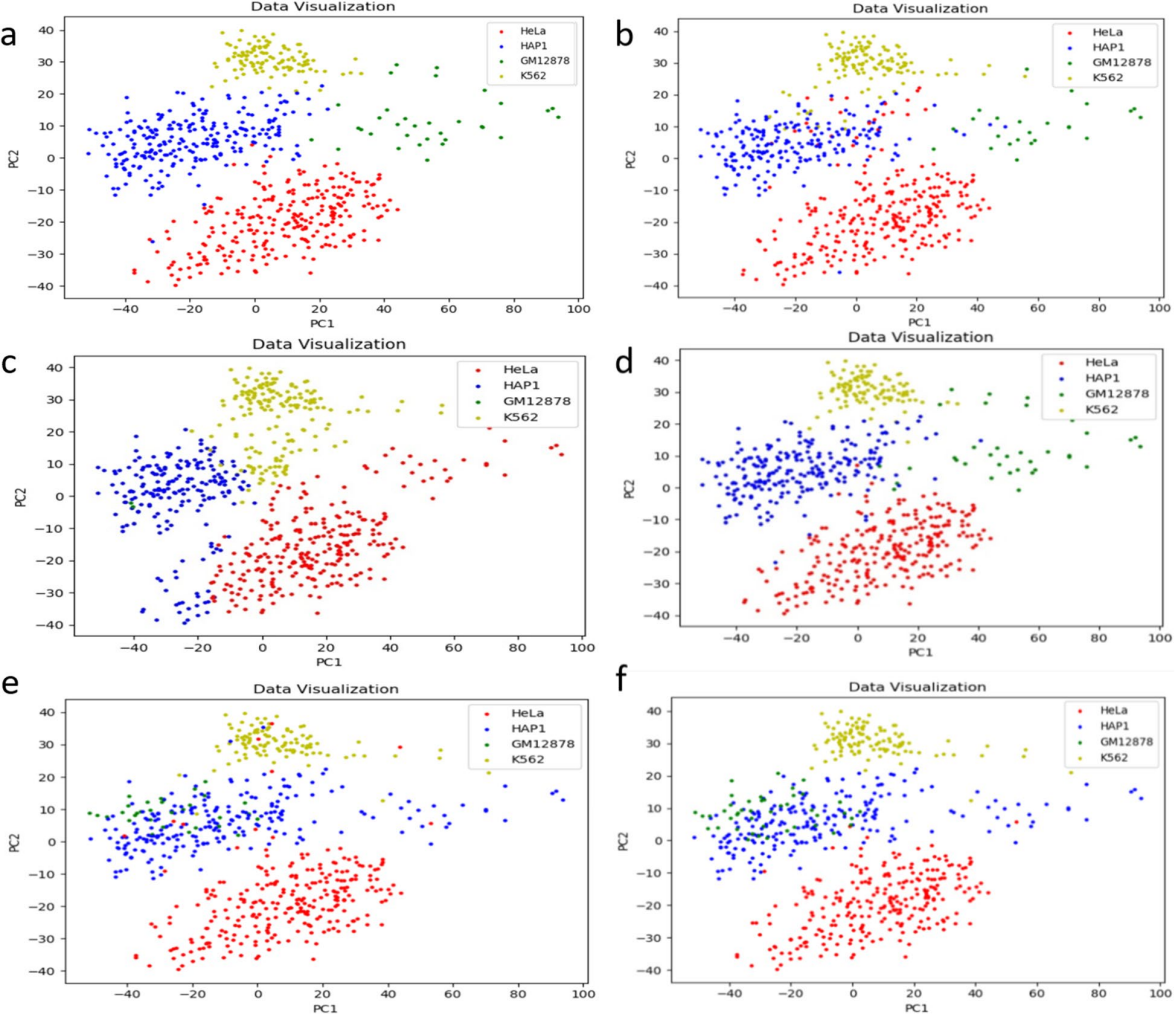
Comparison between SCANN and four other methods on ML1 and ML3 datasets. **a** visualization of results by scHiCluster method. **b** visualization of results by spectral clustering. **c** visualization of results by GMM. **d** visualization of results by hierarchical clustering. **e** visualization of results by SCANN. **f** visualization of real data

As shown in Fig. 5a, d & f, scHiCluster and hierarchical clustering method can correctly identify HeLa cells and K562 cells in ML1 and ML3 datasets, but not able to separate HAP1 cells and K562 cells correctly. As shown in Fig. 5b & f, spectral clustering can correctly identify K562 cells, but not able to clearly distinguish the other three cells. GMM does not correctly divide the four cell types (Fig. 5c & f). However, as shown in Fig. 5e & f, SCANN performs well and can correctly recognize most cell types on ML1 and ML3 datasets.

**Fig. 6:**
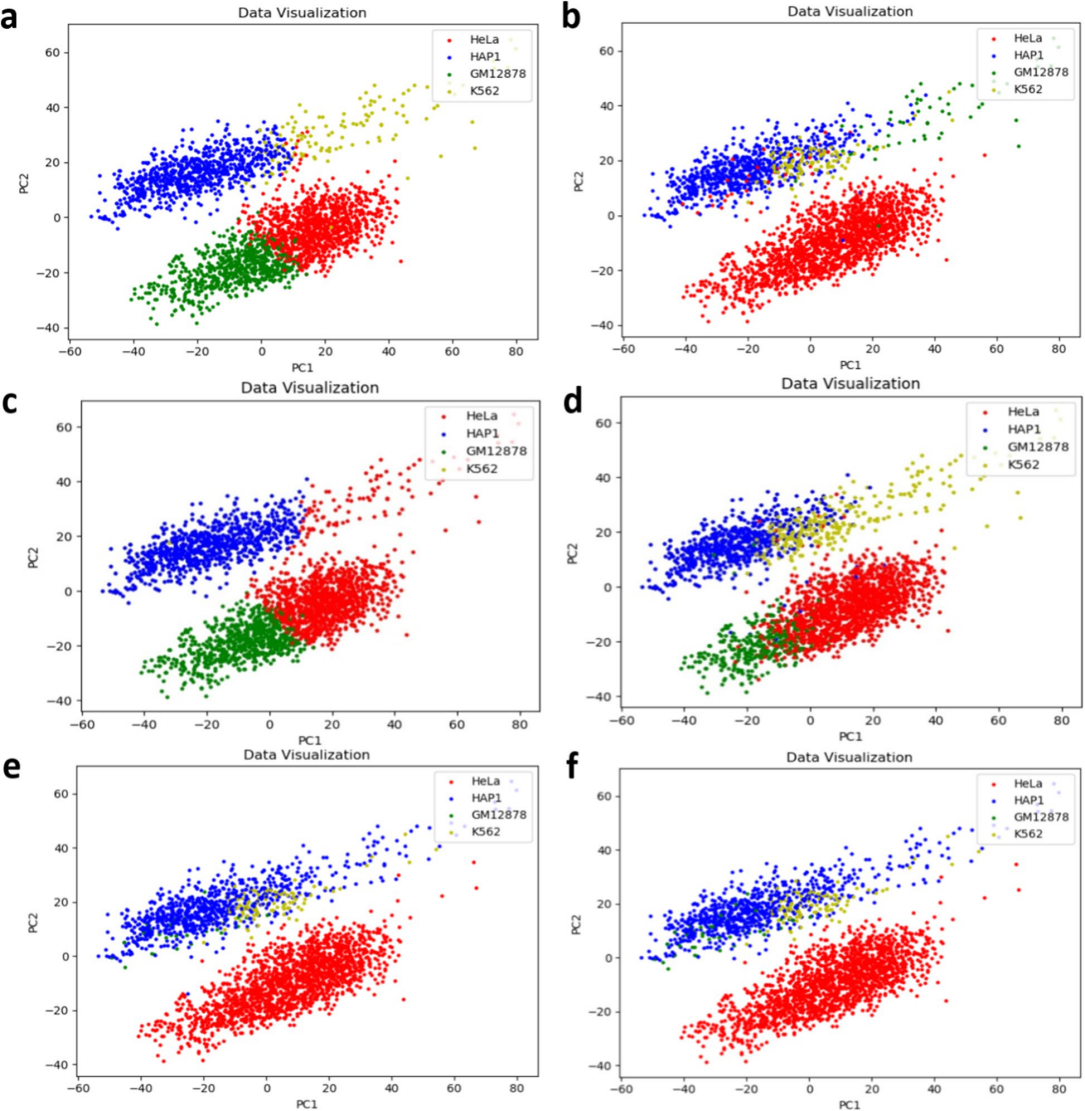
Comparison between SCANN and other four methods on all six libraries of dataset. **a** visualization of results by scHiCluster method. **b** visualization of results by spectral clustering. **c** visualization of results by GMM. **d** visualization of results by hierarchical clustering. **e** visualization of results by SCANN. **f** visualization of real data

Moreover, when used on all six libraries of dataset, scHiCluster and hierarchical clustering methods can identify some HeLa cells as GM12878 cells, and could not distinguish HAP1 cells and K562 cells (Fig. 6a, d & f). As shown in Fig. 6b & f, spectral clustering method can only roughly recognize HeLa and HAP1 cells. GMM could not recognize K562 cells and confused the other three cells (Fig. 6c & f). As shown in Fig. 6e & f, visualizations of the two results are almost the same, indicating that SCANN performs well and can correctly recognize most cell types on all six libraries of dataset. The visualization of result on all six libraries of dataset is better than that just using ML1 and ML3 datasets, indicating that the cell recognition effect of SCANN is better as the number of cells increases.

Thus, our SCANN is robust to the cell quantity distribution of different cell types in the dataset, and the prediction of a few cell types will not be affected by the majority of cells. The increase in the number of cells can improve the performance of the trained model. By recognizing cell types with high accuracy, this method can assist biology researchers to study the dynamic chromosome structure differences between different cell types.

## Discussion

SCANN realizes the correct judgment of input data by stacking full connection layer. This network model can achieve optimal performance with less training times, but there are too many parameters in the model due to the large number of stacked full connection layers. Therefore, it is necessary to construct an large model to complete more complex problems, which is more time-consuming and takes up more memory space.

The SCANN neural network model proposed in this study can recognize cells types with great accuracy as long as the model learn enough representations from training set. In addition, the model has higher stability to the composition of data. However, the model obtained from training dataset can only be used on dataset that is similar to the training dataset because the deep neural networks model is limited by the training dataset. It is therefore necessary to retrain the model with different biological dataset when applying this model in different type of dataset. Even if the internal structure of the model remains unchanged, the model can adapt to different datasets. Additionally, researchers can reset the parameters of the model based on the size of the biological dataset and actual requirements, such as the depth of the neural networks and the number of neurons.

SCANN provides certain extensibility and can be adjusted according to actual requirements and conditions. GPU can also be used to realize parallel acceleration, and researcher can increase the network depth or the number of neurons to improve the model capability. The model can assist biological researchers to study the differences in dynamic chromosomal structures within different cell types, and to combine complex single-cell Hi-C data with powerful computational methods one step further. Our study also broadens the application range of deep learning and validating the advantages of this method.

## Conclusion

Single-cell Hi-C sequencing technology promotes the understanding of cell type heterogeneity and facilitates the analysis of chromosome structure at the single cell level. In this study, we propose a deep neural network model to extract useful information from single-cell Hi-C data to classify cells. We compared the results of our methods with the classical data mining methodology, namely scHiCluster [12]. The analysis shows that our deep neural network approach is more robust than four other methods. And the experimental results show that it is more stable and efficient than four other methods when applied on datasets with uneven cell number distribution. Also, the capability of deep neural network in identifying cell types is higher than four other methods. In order to verify the classification effect of this algorithm, a variety of algorithmic measurement indicators and methods are used, and the analysis shows that our model has high performance on all measures. Compared with the previous methods, the evaluation indexes obtained by our method shows better classification performance. Particularly, SCANN achieves better classification than four other methods when six libraries of dataset are used. Moreover, our method is robust to the sample quantity distribution of different sample types in the dataset, and the prediction of a few sample types is not be affected by the majority of samples.

## Methods

### Data preprocessing

This study is based on cell read-pairs files of combinatorial single-cell Hi-C data from GSE84920 [4]. Four kinds of human cells (GM12828, HAP1, HeLa and K562) from combinatorial single-cell Hi-C data of all six libraries are used in this study. In this analysis, we just focus on intra-chromosomal read-pairs because the dataset can represent an entire chromosome or a continuous chromosome region [12]. For the length of each chromosome L and the preset resolution R, all chromosomes are segmented into nonoverlapping bins, where the number of bins is n=L/R. The contact information of each chromosome is represented as a n$\times$n contact matrix A, where every element of matrix $A_{ij}$ denotes the degree of interaction between the corresponding pair of genomic bins i and j. In this work, the contact matrices are generated at $1-Mbp$ resolution for each chromosome in one cell. An additional filtering step is applied to improve data quality. Specifically, we remove the cells with less than 5k contacts, and also exclude the cells containing at least one chromosome that have no read-pair.

After cell quality control, convolution and random-walk with restarts are used to impute the contact matrices of each chromosome to improve the single-cell Hi-C data quality and resolve data sparsity problem [12]. For each chromosome, we project the matrix into a low-dimensional space and produce the embedding of all chromosomes by Principal Components Analysis (PCA), where the embedding is often applied in the field of natural language processing. In the last step, the embedding of all chromosomes is combined according to the correspondence between chromosome and cell, and another PCA is used to generate the final embedding of each cell. The embedding of one cell can be directly used as the input of the deep neural network.

### Model architecture

**Fig. 7:**
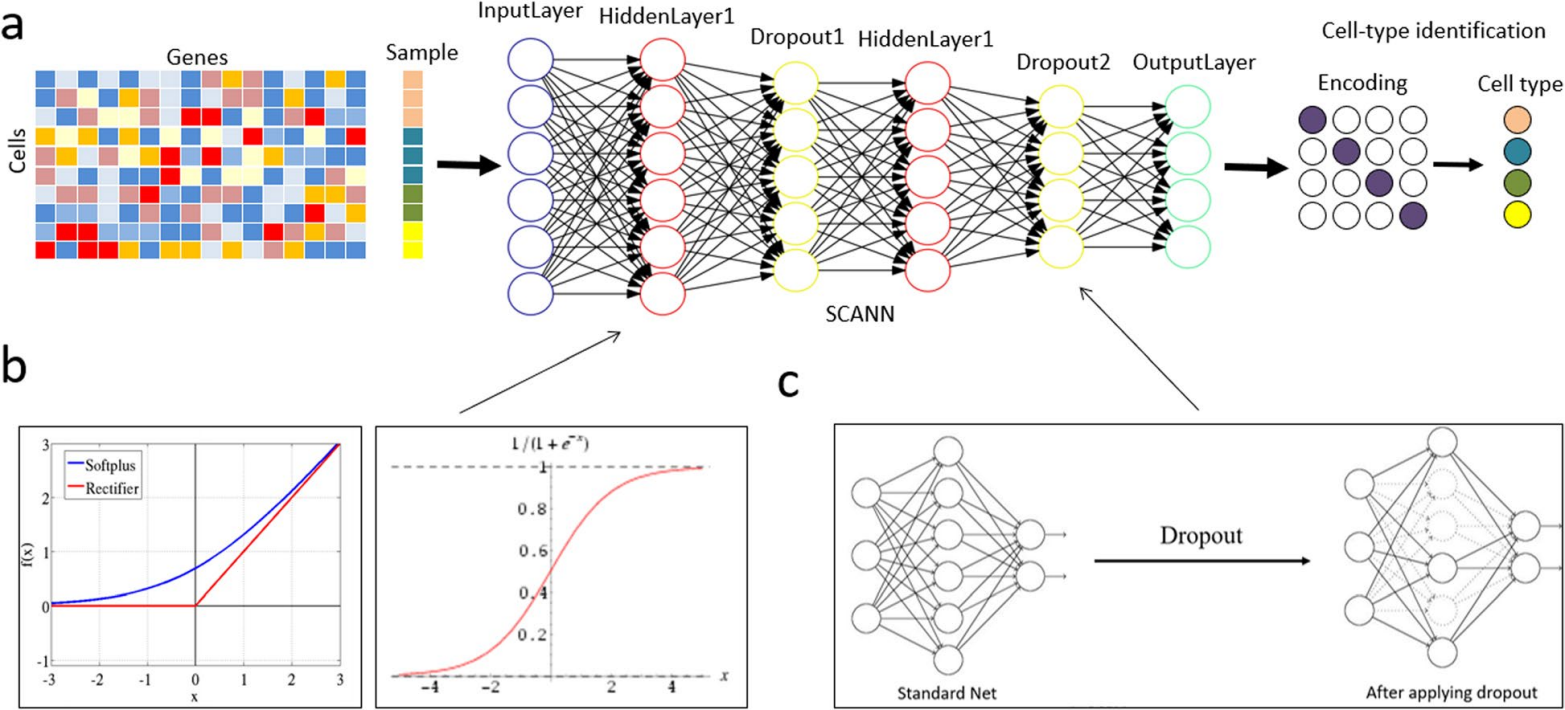
The pipeline of SCANN for classifying single-cell Hi-C data. **a** the structure introduction of SCANN and the overall process of single-cell Hi-C data classification including sample feature matrix input, model training and sample type prediction. **b** ReLU and Sigmoid activation functions used in the model. **c** the dropout regularization interpretation diagram

We propose a deep neural network model to achieve cell classification and cell type prediction, namely single-cell artificial neural networks (SCANN) which can be modified by researcher to flexibly adapt to different datasets. As shown in Fig. 7a, SCANN contains input layer, hidden layer and output layer, in which the number of hidden layers is usually chosen based on the practical requirements. In order to get more accurate results, we apply two hidden layers in SCANN, which can approximate any smooth mapping to any accuracy. The input of network model is set as an interaction matrix with M rows and N columns, in which each row represents one cell and each column represents one feature of each cell. Moreover, the output of network model is a 4-column matrix, in which each row represents each cell and each column represents a kind of cells.

Inside the model, ReLU activation function is applied to the hidden layer of the model, which is useful to solve the convergence problem of deep neural networks, and Sigmoid activation function is used to output the four types of results of the model prediction (Fig. 7b).

In the deep learning networks, if the model has too many parameters and too few training samples, the model is likely to be overfitting after training. Therefore, we utilize Dropout regularization to reduce overfitting of the model due to insufficient data (Fig. 7c). The specific performance of overfitting is as follows: the loss of the model on the training dataset is small, and the prediction accuracy is high. However, on the test dataset, the loss is relatively large and the prediction accuracy is low.

In the training process of deep neural networks, forward propagation is mainly used to realize transformation of nonlinear model from input to output, while back propagation is to optimize mapping capability of nonlinear model, to ensure the predicted value of input data can be as close as possible to the standard output. Therefore, the back propagation takes the loss of the model as the premise, takes the derivative rule of chain as the core, and iteratively updates the model parameters to make the loss continuously reduced as the goal. And the loss function of the model uses the difference between real and predicted values to optimize the objective function. The parameters in the model learn useful data representations and thereby can predict incognizant cells by repeating forward propagation and back propagation.

**Table 3:** The definition of the TP, FP, TN, FN

Reality data	Predicted results	
	true	false
True	TP	FN
False	FP	TN

**Fig. 8:**
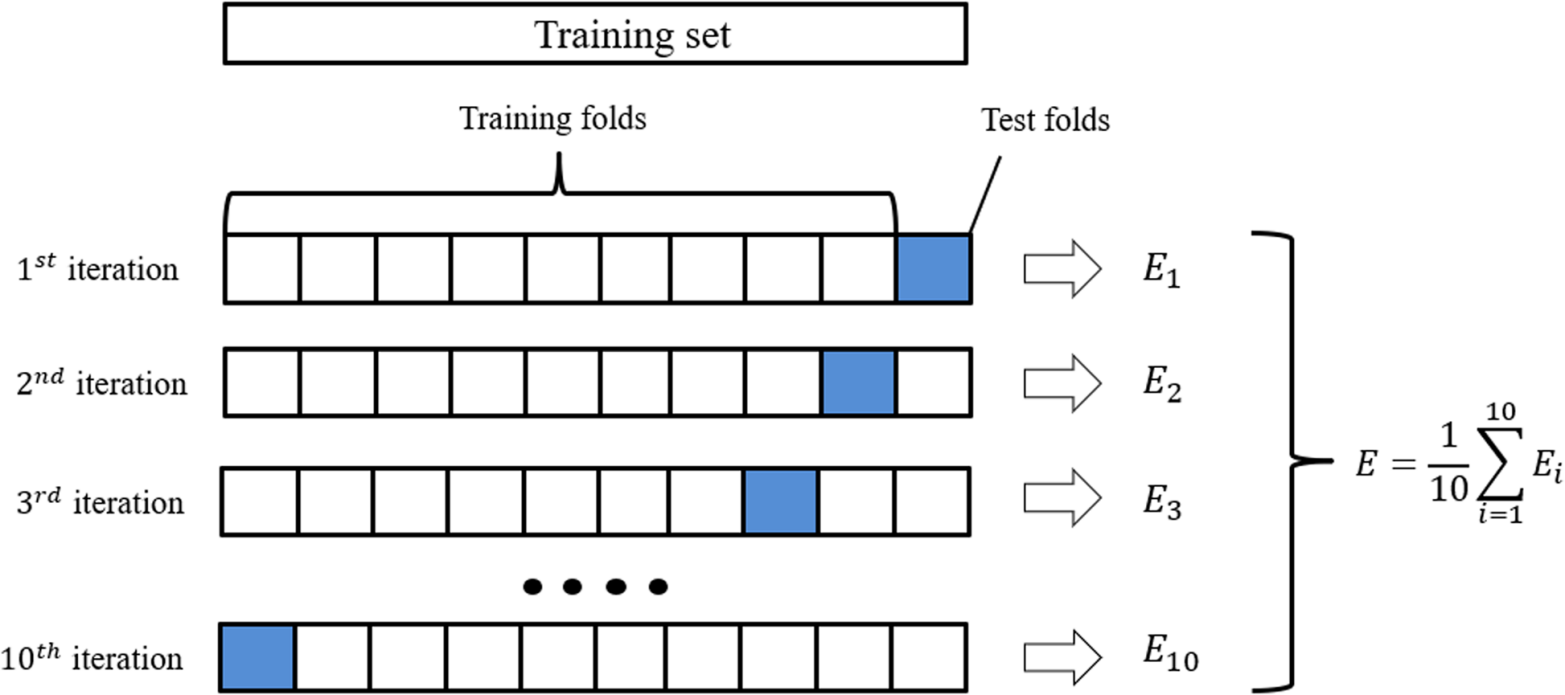
Schematic diagram of cross-validation principle. The calculation process of 10-fold verification index consists of dividing the training set, training and calculating the model verification accuracy rate ten times, and the average value is obtained as cross-validation result of the model

### Measurable indicators

Three indicators are utilized to measure the classification effect of the model on the single-cell Hi-C data, including adjusted rand index (ARI), normalized mutual information (NMI), receiver operating characteristic curve (ROC) and Cross-validation.

#### ARI.

ARI is an effective evaluation index in clustering algorithm, which does not consider the specific implementation and complexity of the clustering algorithm, but only considers the results as a black box [12].

#### NMI.

NMI is used to detect differences between algorithmic classification results and reference results. It has been used to verify the performance of the Incremental Human Action Recognition with Dual Memory (IHAR-DM) algorithm [26]. Given classification result A and reference result B, mutual information (MI) defined in Eq. 1 and entropy of defined in Eq. 2 are used to compute NMI of the A and B by Eq. 3.

1 $$\begin{aligned} MI = \sum \limits _{a\in A}\sum \limits _{b\in B}p(a,b)\cdot \log \left( \frac{p(a,b)}{p(a)\cdot p(b)}\right) . \end{aligned}$$

2 $$\begin{aligned} H(A) = \sum \limits _{a\in A}p(a_i)\cdot \log \left( \frac{1}{p(a_i)}\right) , H(B)= \sum \limits _{b\in B}p(b_i)\cdot \log \left( \frac{1}{p(b_i)}\right) \end{aligned}$$

3 $$\begin{aligned} NMI = 2\cdot \frac{MI}{H(A)+H(B)}. \end{aligned}$$

where *p*(*a*, *b*) is the joint distribution probability of *a* and *b*, and *H*(*A*), *H*(*B*) are the entropy of *a* and *b*, respectively. And, *p*(*a*), *p*(*b*) are the probability function of *a* and *b*, respectively.

#### ROC.

The ROC allows us to visualize the accuracy of an analysis method through diagram, and combines FRP and TPR with graphical method to reflect their relationship accurately. It is a comprehensive representative of the detection accuracy. The ROC curve does not fix the threshold value and allow for the existence of intermediate states, which is conducive for biologists to choose a better threshold value as a diagnostic reference by combining professional knowledge and weighing the impact of missed diagnosis and misdiagnosis. The abscissa and ordinate of ROC curve is false positive rate (FPR) and true positive rate (TPR), which are respectively defined as:

4 $$\begin{aligned} TPR=\frac{TP}{TP+FN}, FPR=\frac{FP}{TN+FP} \end{aligned}$$

where True Positive (TP), False Positive (FP), True Negative (TN) and False Negative (FN) denote the number of samples under four conditions as shown in Table 3. Since it is hard to judge the model when two ROC curves intersect, AUC value is usually used to measure the model’s capability, which is the area covered by ROC curves.

#### Cross-validation.

The Cross-validation method is used to measure two models. There are four cross-validation methods to be released and used, such as Leaveone-out cross-validation (LOOCV), K-fold Cross Validation, Bias-Variance Trade-Off for k-Fold Cross-Validation and Cross-Validation on Classification Problems. Users can adjust the cross-validation scoring strategy for different models and actual scenarios.

We select K-fold cross validation method, and set K to 10. Cross-validation is one effective technique for data scientists in statistical analysis, because it is often necessary to verify the stability of the model and the generalization ability on a new dataset. It needs to ensure that the model trained from the dataset is not affected by noise and has obtained a correct representation of the data. Cross-validation is a model verification technique used to evaluate the generalization ability of a statistical analysis model on independent datasets. As shown in Fig. 8, the dataset without replacement is randomly divided into *K* folds, of which $K-1$ folds are used for training the model, and the remaining fold is used for model performance evaluation. Repeat training and testing the model *K* times to obtain *K* models and their performance evaluation results, and take the average value to obtain the final performance evaluation.

## Data Availability

All data generated or analyzed during this study are included in this published article.

## Abbreviations

Hi-C: High-throughput/resolution chromosome conformation capture
MDS: Multidimensional scaling
SCANN: Single-cell Hi-C artificial neural networks
ROC: Receiver operating characteristic curve
AUC: Area under curve
ARI: Adjusted rand index
NMI: Normalized mutual information
MI: Mutual information
GMM: Gaussian mixture model
HC: Hierarchical clustering
GPU: Graphics processing unit
PCA: Principal components analysis
ReLU: Rectified linear unit
FRP: False positive rate
TPR: True positive rate
TP: True positive
FP: False positive
TN: True negative
FN: False negative
LOOCV: Leave-one-out cross-validation
